# Humidity‐Induced Self‐Oscillating and Self‐Healing Hypercrosslinked Metal–Organic Polyhedra Membranes

**DOI:** 10.1002/advs.202307376

**Published:** 2024-03-11

**Authors:** Jiamin Li, Zhaoyi Liu, Jinjin Liu, Xue Liu, Yang Luo, Jiajie Liang, Zhenjie Zhang

**Affiliations:** ^1^ College of Chemistry Nankai University Tianjin 300071 China; ^2^ School of Materials Science and Engineering National Institute for Advanced Materials Nankai University Tianjin 300350 China; ^3^ State Key Laboratory of Chemical Resource Engineering College of Materials Science and Engineering Beijing University of Chemical Technology Beijing 100029 China; ^4^ Frontiers Science Center for New Organic Matter, Renewable Energy Conversion and Storage Center Nankai University Tianjin 300071 China

**Keywords:** humidity response, metal–organic polyhedra, post‐assembly, self‐healing, self‐oscillating

## Abstract

Designing autonomously oscillating materials is highly desirable for emerging smart material fields but challenging. Herein, a type of hypercrosslinked metal–organic polyhedra (HCMOPs) membranes formed by covalent crosslinking of boronic acid‐modified Zr‐based MOPs with polyvinyl alcohol (PVA) are rationally designed. In these membranes, MOPs serve as high‐connectivity nodes and provide dynamic borate bonds with PVA in hypercrosslinked networks, which can be broken/formed reversibly upon the stimulus of water vapor. The humidity response characteristic of HCMOPs promotes their self‐oscillating and self‐healing properties. HCMOP membranes can realize a self‐oscillating property above the water surface even after loading a cargo that is 1.5 times the weight of the membrane due to the fast adsorption and desorption kinetics. Finally, the HCMOP actuator can realize energy conversion from mechanical energy into electricity when coupled with a piezoelectric membrane. This work not only paves a new avenue to construct MOP‐polymer hybrid materials but also expands the application scopes of MOPs for smart actuation devices.

## Graphical abstract

A new type of hypercrosslinked metal–organic polyhedra (HCMOP) membrane is generated by covalent crosslinking of boronic acid‐modified Zr‐based MOPs with polyvinyl alcohol. Due to the dynamic borate networks, HCMOPs can realize humidity‐induced self‐oscillating and self‐healing properties. The HCMOP actuator realizes energy conversion combined with a piezoelectric membrane and provides a novel platform for the expansion of smart soft materials.

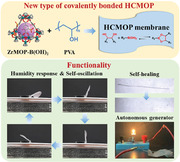

## 1 Introduction

Metal–organic polyhedra (MOPs), as a charming class of crystalline porous materials, have received wide attention and have been regarded as perfect candidates for the frontier of applications, due to their definite structure and intrinsic porosity.^[^ ^1^, ^2^, ^3^ ^]^ More importantly, MOPs have solution processability, which can differ from other porous crystalline materials such as metal–organic frameworks or zeolites.^[^ ^4^, ^5^ ^]^ Over the past decade, many studies have been devoted to the functional modification of MOPs so that MOPs can be decorated with a wide variety of functional groups by one‐step synthesis or post‐synthetic modification, not only facilitating the establishment of an extensive library of MOPs but also introducing pre‐organized subunits within extended materials, to further form some functional materials with processability, such as gels or membranes.^[^ ^6^, ^7^ ^]^ In addition, with the combination of modified MOPs and other components through numerous interactions (e.g., covalent bonds, coordination bonds, hydrogen bonding interaction, electrostatic interaction, and host–guest interaction), the physical and chemical properties of the extended materials are improved and new functions are generated.^[^ ^8^, ^9^, ^10^ ^]^

Surpassing the traditional physical mixing strategy, the covalent binding strategy of MOPs with polymers can further overcome the shortcomings of poor mechanical properties and weak binding forces between inorganic fillers and polymers.^[^ ^11^ ^]^ However, the covalent crosslinking between MOPs and polymers not only requires MOPs to have excellent solubility, stability, and high reactivity but also the appropriate solvent systems, high chemical selectivity, and mill reaction conditions are prerequisites. So far, the reported crosslinking reactions of MOPs focus on the condensation reaction of the amino group and aldehyde group/acyl chloride, the olefin polymerization, the click reaction, and the amination reaction.^[^ ^12^, ^13^, ^14^, ^15^, ^16^, ^17^, ^18^, ^19^, ^20^, ^21^ ^]^ The main reason for the lack of covalent crosslinking bond types is the harsh reaction conditions of many covalent reactions, such as high temperature, strong acid or base systems, and even additional catalysts. The relatively weak coordination bond of MOPs obviously cannot withstand some harsh reaction environments. The limited reaction type further limits the application range of MOPs. These requirements put forward higher standards for the reactivity and stability of MOPs. Therefore, designing and developing new MOPs and their new types of covalent crosslinking reactions are very meaningful, which not only can expand the systems of MOP‐polymer hybrid material, but also provide novel ideas for developing the applications of MOPs and polymers.

**Scheme 1:**
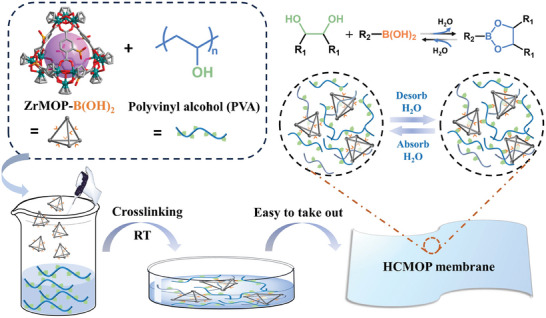
Illustration of constructing dynamic humidity‐responsive HCMOP membranes via covalently crosslinking boric acid‐modified ZrMOP with PVA.

In this work, we designed and synthesized a new boronic acid‐modified Zr‐MOP and prepared dynamic hypercrosslinking MOPs (HCMOPs) network based on covalent crosslinking with polyvinyl alcohol (PVA) (**Scheme** **1**). To our knowledge, the polymer‐MOP hybrid material linked by borate bond has not been reported. Compared to other bonding types, boronate ester bond is a kind of dynamic covalent bond, that is capable of exchanging bond connectivity and undergoing instantaneous debonding and rebonding at ambient conditions. Boronic acid‐modified MOPs had strong hydrophilicity, and the dynamic borate ester bond formed by crosslinking with PVA enhanced the humidity response of the HCMOPs membrane. Moreover, the combination of rigid structure and flexible polymer could significantly improve the mechanical performance and the response‐ability of the HCMOPs membrane. The HCMOPs membrane could oscillate on the humidity gradient spontaneously formed on the water surface. Based on the constructed HCMOP membrane, we further couple it with piezoelectric polyvinylidene fluoride (PVDF) to prepare a self‐oscillation generator. The new type of HCMOPs demonstrates a new kind of humidity‐responsive smart materials and expands the application scopes of MOPs.

## 2 Results and Discussion

### 2.1 Design and Synthesis of ZrMOP

**Figure 1:**
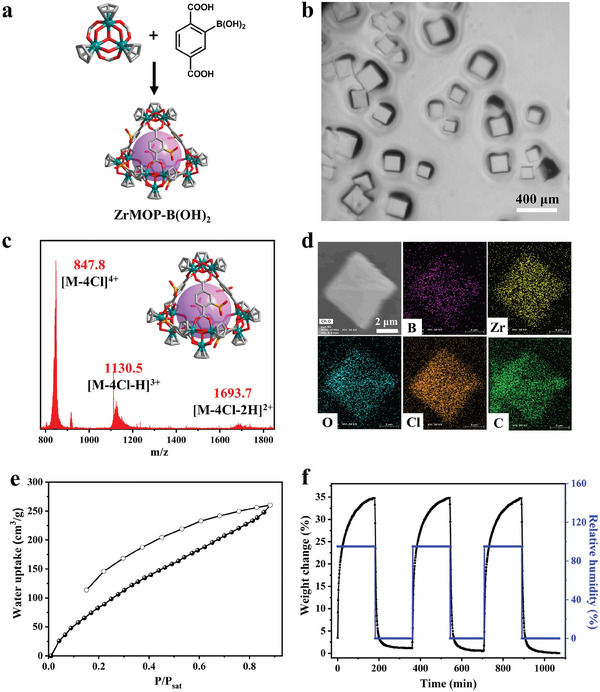
a) Constructing ZrMOP‐B(OH)_2_ from Cp_3_Zr_3_(*µ*_3_‐O)(*µ*_2_‐OH)_3_ SBUs and H_2_BDC‐B(OH)_2_. Zr, dark green; C, gray; O, red; B, yellow. H atoms and uncoordinated Cl ions were omitted for clarity. The purple ball represents the cavities of MOPs. b) Optical microscope photo of ZrMOP‐B(OH)_2_ crystals. c) UPLC‐Q‐TOF‐MS analysis of ZrMOP‐B(OH)_2_ in methanol/H_2_O. d) EDX mapping of ZrMOP‐B(OH)_2_ crystal. e) Water vapor adsorption isotherm of ZrMOP‐B(OH)_2_ at 298 K. Solid circles = adsorption, empty circles = desorption. f) Dynamic vapor adsorption‐desorption curves of ZrMOP‐B(OH)_2_ over 3 cycles.

Zr‐based MOPs^[^ ^22^, ^23^ ^]^ usually possess excellent water stability and solubility, and boronic acids can form dynamic covalent bonds with *cis*‐diols to construct dynamic boronate esters‐crosslinked networks.^[^ ^24^, ^25^ ^]^ Thus, we rationally designed and synthesized a new boronic acid‐modified Zr‐based MOP, called ZrMOP‐B(OH)_2_. 2‐boronoterephthalic acid (H_2_BDC‐B(OH)_2_) was dissolved together with bis(cyclopentadienyl)zirconium dichloride (Cp_2_ZrCl_2_) in a mixture of N,N‐dimethylacetamide (DMA) and water, followed by heating at 60 °C for 8 h to afford ZrMOP‐B(OH)_2_ as colorless cubic crystals (**Figure** **1**a,b). Due to the poor X‐ray diffraction ability of the crystal, the single crystal data of ZrMOP‐B(OH)_2_ to fully solve its structure cannot be obtained. Fortunately, the unit cell parameters (*a* = *b* = *c* = 36.578 Å, *α* = *β* = *γ* = 90°) with *Fm*3__*m* symmetry of ZrMOP‐B(OH)_2_ were obtained, which matched well with the unit cell parameters of reported Zr‐based tetrahedral MOPs.^[^ ^26^, ^27^ ^]^ To further verify the structure of ZrMOP‐B(OH)_2_, the reported methyl‐modified tetrahedral ZrMOP was selected, here named ZrMOP‐CH_3_ (also refer to MOC‐QW‐2‐CH_3_^[^ ^27^ ^]^). The powder X‐ray diffraction (PXRD) (Figure S1, Supporting Information) patterns of the as‐synthesized ZrMOP‐B(OH)_2_ crystals agreed well with the simulated PXRD pattern of ZrMOP‐CH_3_, indicating that ZrMOP‐B(OH)_2_ had the same tetrahedral cage structure as ZrMOP‐CH_3_. The structure of ZrMOP‐B(OH)_2_ was further confirmed by ^1^H NMR and ultra‐performance liquid chromatography‐quadrupole mass spectrometry (UPLC‐Q‐TOF‐MS). The ^1^H NMR spectra showed 6.6 and 8.1 ppm resonance signals, which demonstrated the presence of ZrMOP‐B(OH)_2_ (Figure S2, Supporting Information). In addition, the UPLC‐Q‐TOF‐MS results observing ion peaks at m/z of 847.8, 1130.5, and 1693.7 correspond to the [M‐4Cl]^4+^, [M‐4Cl‐H]^3+^, and [M‐4Cl‐2H]^2+^ ions, respectively, also provided support for the presence of intact cationic tetrahedral cages in solution (Figure 1c). Energy‐dispersive X‐ray (EDX) mapping analysis verified the presence of all elements in ZrMOP‐B(OH)_2_, especially the boron element (Figure 1d).

The porosity of ZrMOP‐B(OH)_2_ was determined by N_2_ adsorption analysis at 77 K. ZrMOP‐B(OH)_2_ exhibited a type‐I isotherm (Figure S3, Supporting Information), with a Brunauer–Emmett–Teller surface area of 310 m^2^ g^−1^ and Langmuir surface area of 497 m^2^ g^−1^. Water vapor sorption tests and dynamic water adsorption (DVS) were used as a preliminary assessment of the amount and rate of water vapor adsorption by ZrMOP‐B(OH)_2_. Specifically, ZrMOP‐B(OH)_2_ had a maximum water uptake of 260.2 cm^3^ g^−1^ at 298 K attributed to the high porosity and abundant hydrophilic groups (e.g., boric acid groups) (Figure 1e). The DVS result revealed that ZrMOP‐B(OH)_2_ could absorb water in the airflow (34.5% weight increase) within 160 min when the airflow humidity switched from 0% RH to 95% RH (Figure 1f). When the airflow humidity dropped to 0% RH, ZrMOP‐B(OH)_2_ could recover to its original weight (i.e., release all adsorbed water) within 22 min. The desorption rate was faster than the absorption rate. Overall, ZrMOP‐B(OH)_2_ had stronger water adsorption capacity and fast desorption kinetics that were instructive for the further study of the humidity response of the HCMOP membrane.

### 2.2 Preparation and Characterization of Membranes

**Figure 2:**
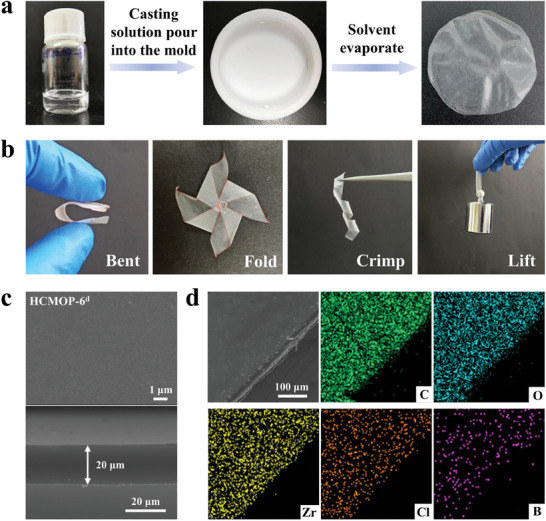
a) The preparation process of HCMOP‐6 membrane; b) Mechanical performance of HCMOP‐6 membranes. (The red stroke on the edge of the membranes in the picture was only for the clearer display.) c) Top view (top) and cross‐section (bottom) SEM images of the freestanding HCMOP‐6^d^ membrane. d) EDX mapping of HCMOP‐6^d^ membrane.

(PVA is a polymeric material with good biocompatibility, outstanding film‐forming ability, excellent hydrophilicity, and a large number of hydroxyl groups that can react with boronic acid functional groups.^[^ ^28^, ^29^ ^]^ A condensation reaction was employed to prepare the MOP‐polymer network (named HCMOP‐6) via ambient polymerization of PVA and ZrMOP‐B(OH)_2_ in mixed organic solvents of N,N‐dimethylformamide (DMF) and water at room temperature. To promote polymerization, triethylamine (NEt_3_) was added to the prepolymer solution. Then the clear solution was poured into a polytetrafluorocarbon (PTFE) mold. A uniformly and freestanding HCMOP‐6 membrane was obtained as the evaporate solvents. In HCMOP‐6, ZrMOP‐B(OH)_2_ was used as hypercrosslinking 6‐connected nodes to react with the hydroxyl groups of PVA chains, thus forming dynamic boronic esters in the crosslinking network. A series of HCMOP‐6 membranes were fabricated by adjusting the loading of MOPs (a = 1/36, b = 1/18, c = 1/15,  = 1/12, representing the molar ratio of ZrMOP‐B(OH)_2_/PVA) (**Figure** **2**a; Figure S4, Supporting Information). It was found that when further increasing the ratio of ZrMOP‐B(OH)_2_/PVA to 1/9, uniformed membranes could not be produced, and MOP crystals gradually precipitate in the membrane (Figure S5, Supporting Information). It was because the excessive MOPs could not participate in the polymerization reaction, leading to further aggregation and assembly during solvent evaporation and sample drying.

**Figure 3:**
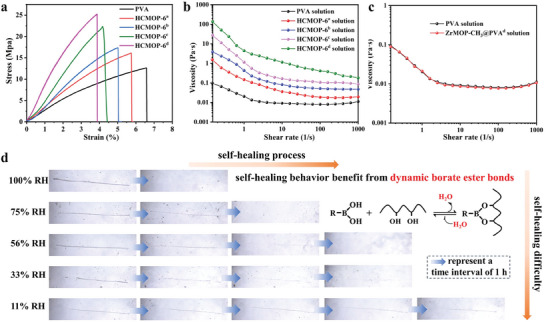
a) Stress−strain curves of dry PVA and HCMOP‐6 membranes. b) Shear sweep of PVA and HCMOP‐6 solutions (The solvent volume was fixed at DMF/H_2_O = 1.5 mL/0.45 mL and the crosslinking time was fixed at 30 min). c) Shear sweep of PVA and ZrMOP‐CH_3_@PVA^d^ solutions (The solvent volume was fixed at DMF/H_2_O = 1.5 mL/0.45 mL and the mix tim was fixed at 30 min). d) Self‐healing behavior of HCMOP‐6^d^ membrane under different humidities.

The uniform and freestanding HCMOP‐6 membranes possessed good quality and mechanical performance and could be bent, folded, curled, and loaded (Figure 2b). The microstructure and uniformity of HCMOP‐6 and PVA membranes were observed by scanning electron microscopy (SEM) and EDX mapping analysis. As shown in SEM images (Figure 2c; Figure S6, Supporting Information), all HCMOP‐6 membranes possess smooth surfaces and compact packing without defects on the surface and cross sections. EDX mapping (Figure 2d; Figure S7, Supporting Information) revealed that ZrMOP‐B(OH)_2_ was homogeneously dispersed throughout the membranes. Mechanical properties of PVA and HCMOP‐6 membranes were investigated by stress−strain experiments. As shown in **Figure** **3**a, HCMOP‐6 membranes have significantly enhanced tensile strength and Young's modulus compared to PVA. With an appropriate increase in the amount of MOPs, the tensile strength and Young's modulus of HCMOP also increase. Because each ZrMOP‐B(OH)_2_ as the high‐connectivity nodes could provide six reactive sites, the tensile strength, and Young's modulus in HCMOPs increased with higher crosslinking density. Among them, HCMOP‐6^d^ showed the highest Young's modulus of 815 MPa, almost 5.2 times as much as the pure PVA membrane (156 MPa) (Table S1, Supporting Information). In Fourier transform infrared (FT‐IR) spectra (Figure S8, Supporting Information), the ─C─H and ─C─O characteristic peaks of PVA and ─B─O characteristic peaks of ZrMOP‐B(OH)_2_ could be identified by the FT‐IR spectra of the HCMOP‐6.^[^ ^30^ ^]^

Considering the stability of ZrMOP‐B(OH)_2_ during the crosslinking process, the UPLC‐Q‐TOF‐MS was used to investigate the effect of reaction systems on the stability of ZrMOP‐B(OH)_2_. As shown in Figure S9 (Supporting Information), ZrMOP‐B(OH)_2_ remained intact in the DMF/water system or the presence of NEt_3_ (amount of reaction). Small‐angle X‐ray scattering (SAXS) was applied to further probe the morphologies and dispersion of ZrMOP‐B(OH)_2_ in HCMOP‐6 membranes (Figure S10, Supporting Information). The form factor of ZrMOP‐B(OH)_2_ was found which suggested the intactness of the ZrMOP‐B(OH)_2_ structure. In the low‐*q* range (0.11–0.15 Å^−1^) of SAXS data, the resolved broad peak could be assigned to the structure factor of ZrMOP‐B(OH)_2_ and indexed as the average distances (*d*, inversely proportional to the *q*) between neighboring MOPs in the polymer matrix. With the increase of MOPs amount, the average distances gradually decreased, indicating the increase of crosslinking degree.

To verify the formation of the covalent bond between MOPs and PVA, the shear sweep measurement on the rheometer was carried out to evaluate the viscosity of the solution gradually changed with increasing crosslinking densities.^[^ ^31^ ^]^ It was found that the viscosity of the HCMOP‐6 solution increased with the increase of ZrMOP‐B(OH)_2_ content, and all solutions demonstrated shear thinning with the chains aligned at high shear rates (Figure 3b). To further prove the covalent crosslinked between ZrMOP‐B(OH)_2_ and PVA, we synthesized ZrMOP‐CH_3_.^[^ ^27^ ^]^ The PXRD of the as‐synthesized colorless cubic crystals (Figure S11, Supporting Information) and the UPLC‐Q‐TOF‐MS analysis in methanol (Figure S12, Supporting Information) indicated that ZrMOP‐CH_3_ has successfully synthesized. Then, a series of mixed matrix membranes, ZrMOP‐CH_3_@PVA, was prepared with the same preparation process as HCMOP‐6. The additional amount of ZrMOP‐CH_3_ was consistent with ZrMOP‐B(OH)_2_ in HCMOP‐6. ZrMOP‐CH_3_ without boronic acid functional groups cannot be covalently crosslinked with PVA, therefore it can only be physically dispersed in the polymer matrix. Compared to PVA, ZrMOP‐CH_3_@PVA membranes showed poorer mechanical properties, because of the lack of strong interaction between MOPs and PVA and the inevitable aggregation of ZrMOP‐CH_3_ (Figure S13, Supporting Information). SEM images also confirmed that the ZrMOP‐CH_3_@PVA^d^ membrane, similar to the traditional mixed matrix membrane, possessed distinguishable defects or voids (Figure S14, Supporting Information). The shear sweep measurement of ZrMOP‐CH_3_@PVA^d^ was also conducted as a comparison. The viscosity of the ZrMOP‐CH_3_@PVA^d^ solution was almost the same as the PVA solution due to the absence of covalent bond crosslinking by MOPs and polymer (Figure 3c). In addition, a model molecule (1) was synthesized to confirm covalent bonding between ZrMOP‐B(OH)_2_ and PVA.^[^ ^32^ ^]^ Phenylboric acid reacted with propylene glycol under conditions consistent with the fabrication of HCMOP‐6. ^1^H NMR data verified the reaction by the appearance of new peaks of 3.7, 4.3, and 4.6 ppm (Figure S15, Supporting Information). Furthermore, model molecule (2) was synthesized through a reaction between ZrMOP‐B(OH)_2_ and propylene glycol in the same synthesis condition as HCMOP‐6^d^. ^1^H NMR data confirmed the formation of borate bonds by the appearance of new peaks of 3.8 and 4.05 ppm. These two peaks disappeared upon the addition of D_2_O, suggesting the dynamic nature of the boronate ester bond (Figure S16, Supporting Information).

### 2.3 Self‐Healing Behavior and Solution Processability

Owing to the dynamic nature of reversible boronate ester bonds and polymer chain movement, HCMOP‐6 membranes showed interesting self‐healing ability.^[^ ^33^, ^34^, ^35^ ^]^ For instance, HCMOP‐6^d^ membranes were cut with a 2 mm scratch by a surgical blade at room temperature. After the membranes with cracks were placed into a series of humidity environments (except for 100% RH, other humidities were achieved by different salt solutions: LiCl (11% RH), MgCl_2_ (33% RH), Mg(NO_3_)_2_ (56% RH), NaCl (75% RH)) shown in Figure 3d, as humidity increased, the self‐healing time became shorter. The HCMOP‐6^d^ membrane could achieve complete self‐healing at RH > 33%, verified by SEM and stress−strain results (Figures S17 and S18, Supporting Information). The higher the humidity, the faster the dynamic conversion between boric acid and its ester derivatives, thereby promoting the bond reformation at the crack.

Taking advantage of the dynamic feature of the boronate ester bond in the crosslinked network, HCMOP‐6 membranes possessed excellent solution processability. We chose HCMOP‐6^d^ with the highest crosslinking degree as the representative. HCMOP‐6^d^ was cut into small pieces and fully dissolved in DMF/H_2_O mixed solution under heating at 60 °C. Then the polymer solution could be refabricated to an integrated membrane (Figure S19, Supporting Information). Therefore, whether surface cracks or overall damage, HCMOP‐6 can achieve complete healing, thereby extending its service life.

### 2.4 Self‐Oscillating Behavior and Study of Its Affecting Factors

**Figure 4:**
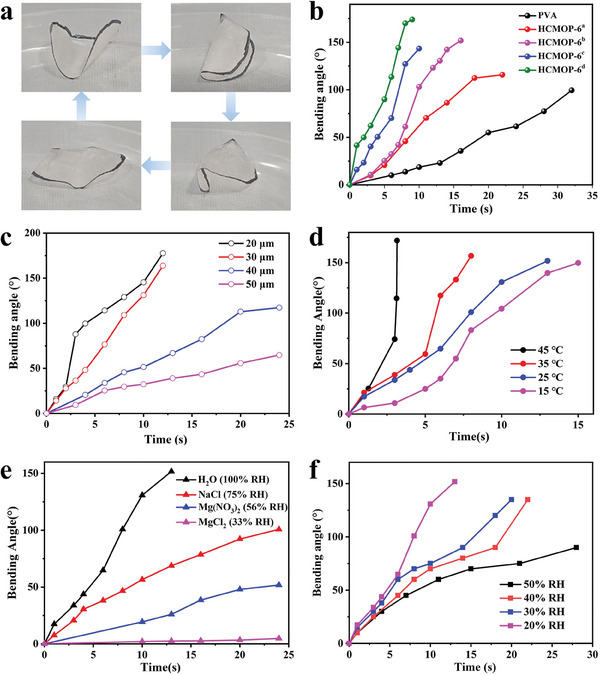
a)  Representative images of the continuous flipping process of HCMOP‐6^d^ above the water surface at 25 °C. (The black stroke on the edge of the membranes in the picture was only for the clearer display). b) Comparison of bending angle of HCMOP‐6 with different loading amounts above the water surface (thickness: 20 µm, ambient environment: 25 °C, ≈18% RH, water temperature: 25 °C). c) Comparison of bending angle of HCMOP‐6^d^ with different thicknesses above the water surface (ambient environment: 25 °C, ≈20% RH, water temperature: 25 °C). d) Comparison of bending angle of HCMOP‐6^d^ with different water temperatures above the water surface (thickness: 20 µm, ambient environment: 25 °C, ≈20% RH). e) Comparison of bending angle of HCMOP‐6^d^ with different humidity stimulation (thickness: 20 µm, ambient environment: 25 °C, water temperature: 25 °C). f) Comparison of bending angle of HCMOP‐6^d^ with different ambient humidity above the water surface (thickness: 20 µm, water temperature: 25 °C).

PVA, a hydrophilic polymer, possesses good hygroscopicity and undergoes volume expansion after water absorption.^[^ ^36^ ^]^ The dynamic boronic ester bonds can undergo dynamic bond exchange in humidity conditions.^[^ ^37^ ^]^ Hence, the HCMOP‐6 membranes could show humidity‐induced macroscopic dynamic behavior when exposed to a certain humidity gradient. Thereupon, the humidity‐induced actuation behavior of HCMOP membranes was qualitatively investigated at room temperature.^[^ ^38^ ^]^ It could be seen that HCMOP membranes could continuously flip, and the flipping process could be divided into three steps when placed on the porous nylon mesh with water vapor underneath (**Figure** **4**a; Video S1, Supporting Information). I) The bottom surface of the membrane, first, contacted more water vapor than the top surface, as the boronic ester bond broke and the volume expansion of the bottom surface of the membrane, both sides of the membrane upward bent; II) With the upward bending of HCMOP membrane, the contact area between the membrane and the substrate became smaller resulting in the unstable center of gravity and membrane falling to one side; (III) With the membrane continued to bend, the dehydration and water absorption on membrane surface were exchanged causing the membrane to flip over. It should be emphasized that the actuation of HCMOP‐6 has unidirectionality, that is, it moves in a direction away from the water surface. Because the lower surface near the water vapor side absorbs more water molecules to expand rapidly, while the upper surface absorbs fewer water molecules to expand slowly. The generation of the humidity gradient is the fundamental cause of membrane movement.

Then, we secured the side of the membrane materials on the water or other salt solution surface at ≈25 °C and systematically studied the relationship between the bending properties of the HCMOP membrane and various factors such as the loading of ZrMOP‐B(OH)_2_, membrane thickness, water temperature, and humidity. The definition and measurement of the bending angle are shown in Figure S20 (Supporting Information).^[^ ^39^, ^40^ ^]^ The HCMOP membranes were clamped and exposed to moisture from one end. As illustrated in Figure 4b, compared with pure PVA membrane, HCMOP‐6^d^ had the fastest response speed with the shortest activation process above the water surface, indicating that adding ZrMOP‐B(OH)_2_ improved the humidity sensitivity. An increase of ZrMOP‐B(OH)_2_ loading from a to d significantly increases the humidity sensitivity, which was reflected in the faster bending response and recovery speed (Figure S21, Supporting Information). This may be because the density of the boronic ester bond and the membrane's mechanical properties increased with the increase of ZrMOP‐B(OH)_2_ loading, promoting the humidity response of the membrane. HCMOP‐6^d^ could achieve a bending angle of *θ* = 178° in as short as 10 s after exposure to water vapor and could rapidly recover back to the *θ* = 22° position once removed within 18 s (Figure S22, Supporting Information).

Different thicknesses of membrane actuators should show a great influence on their own water absorption and desorption rate. As shown in Figure 4c, the optimal thickness for the bending response of the membrane was 20 µm. With the thickness increasing from 20 to 50 µm, the rate of water absorption and desorption decreased and the time required for a complete water absorption and dehydration process increased from 13 to 25 s (Figures S23 and S24, Supporting Information). The bending speed of HCMOP‐6^d^ also varies with the temperature. It was found that the higher the temperature, the faster the bending speed (Figure 4d). Because HCMOP‐6^d^ has strong water sensitivity, it can respond quickly at low temperatures (e.g., 25 °C). We further studied the influence of humidity gradients on humidity‐responsive performance by changing the humidity source below the membrane or the ambient humidity, respectively. A series of humidity sources below the membrane was achieved using different salt solutions (MgCl_2_ (33% RH), Mg(NO_3_)_2_ (56% RH), NaCl (75% RH)). As shown in Figure 4e,f, the larger the humidity gradient on both sides of the membrane, the faster the response speed and the greater the bending angle of the membrane. In addition, the humidity‐induced bending behaviors of HCMOP‐6^d^ before and after self‐healing were studied. The results showed that there was no significant change in the bending angle of pristine HCMOP‐6^d^ and the sample after self‐healing, indicating that the sample can completely self‐heal without affecting the humidity response characteristic (Figure S25, Supporting Information).

**Figure 5:**
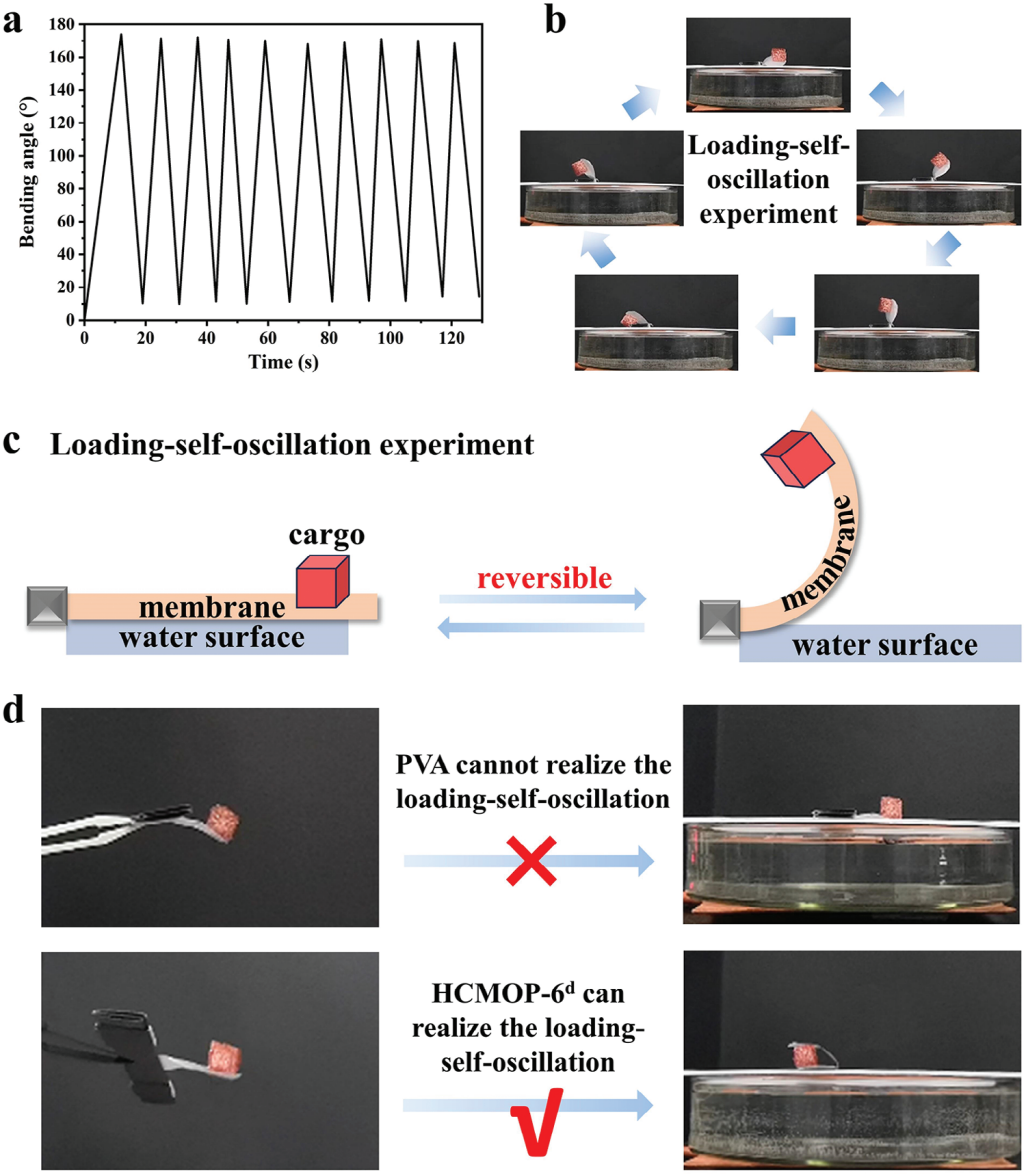
a) The self‐oscillation process of HCMOP‐6^d^ underwent 10 cycles upon moisture gradients on/off switches (thickness: 20 µm, ambient environment: 25 °C, ≈20% RH, water temperature: 25 °C). b) Photographs of the loading‐self‐oscillation experiment of HCMOP‐6^d^ above the water surface (thickness: 20 µm, ambient environment: 25 °C, ≈20% RH, water temperature: 25 °C). c) Schematic diagram of loading‐self‐oscillation experiment. d) The loading‐self‐oscillation experiment comparison between PVA and HCMOP‐6^d^ (thickness: 20 µm, ambient environment: 25 °C, ≈20% RH, water temperature: 25 °C).

With the help of the humidity gradient formed spontaneously above the water surface, the HCMOP membrane could continuously self‐oscillate above the water surface without any external interference (Figure S26 and Video S2, Supporting Information).^[^ ^41^ ^]^ The bending and recovery process was repeatable over 10 times upon moisture gradients on/off switches (**Figure** **5**a). As the number of cycles increased, the membrane response speed became faster because the membrane underwent an activation process above the water surface. When one end of the membrane was clamped and the other end was loaded with a weight, named loading‐self‐oscillation experiment, its movement was the same as the above process (Figure 5b; Video S2, Supporting Information). As shown in Figure 5b,c, when one end of the membrane was fixed with a cargo that was 1.5 times its weight, the response time was 100 s for 5 cycles of oscillation, which was slower than unloading because of increasing the process of overcoming the weight of the cargo (Figure S27, Supporting Information). HCMOP‐6^d^ could lift the heaviest cargo that was 10 times as heavy as itself with a bending angle of nearly 170° (Figure S28, Supporting Information). Unfortunately, after removing the water source, the dehydration recovery force of HCMOP‐6^d^ made it unable to overcome the gravity of the cargo and return.

The loading‐self‐oscillation experiment was used to judge the work capacity of the membranes, which was an important indicator for judging the humidity response performance of membranes. HCMOP‐6^d^ could achieve the loading‐self‐oscillation (Figure 5d), indicating that the introduction of ZrMOP‐B(OH)_2_ not only increased the crosslinking density of the membrane, thereby increasing the strength of the membrane but also accelerated the response speed of the membranes. However, under the same conditions, the PVA could not realize the loading‐self‐oscillation due to the PVA lacking rigidity and the response speed of PVA was slow. With the extension of time, the surface of PVA will absorb water and become soft, which makes PVA lack support and do no work. The HCMOP‐6^d^ membrane could carry a certain mass of cargo to move together, which laid a foundation for the subsequent composite with piezoelectric materials to convert mechanical energy into electrical energy.

### 2.5 Energy Conversion and Humidity Detection

**Figure 6:**
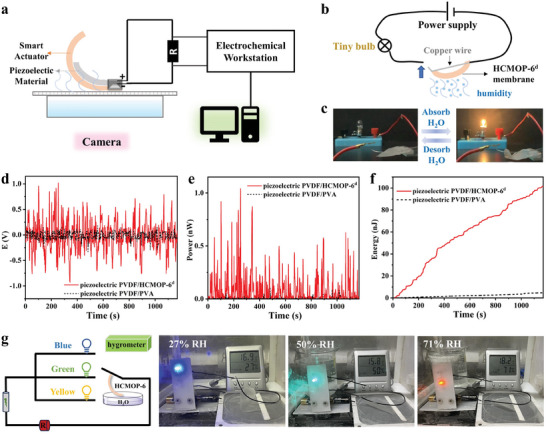
a) Schematic diagram of generator assembly and operation. b,c)  Electric circuit (b) and photograph for lighting small bulb (c). d–f) Voltage (d), instantaneous power (e), and instantaneous energy (f) output of piezoelectric PVDF/HCMOP‐6^d^ and PVDF/PVA self‐oscillating generator under humidity stimulation. g) The circuit diagram of the membrane‐based humidity switch and the response of the LED lights under different ambient humidity.

The self‐oscillation and fast response properties allowed the HCMOP membrane to serve as an actuator promising for application in the energy harvesting field.^[^ ^42^, ^43^, ^44^ ^]^ As shown in **Figure** **6**a and Video S3 (Supporting Information), a generator was designed and built using a combination of HCMOP‐6^d^ and piezoelectric elements (PVDF membrane metalized by aluminum). Specifically, the PVDF was placed on the HCMOP membrane and driven with the self‐oscillating HCMOP‐6^d^ above the water surface without introducing extra operations, converting mechanical energy into electrical energy.^[^ ^45^ ^]^ Moreover, the anti‐deformation property of the PVDF can promote the recovery of the bending of the HCMOP membrane to some extent. As a result, the energy accumulated in the resistor in 20 min outputting a peak voltage reached −0.93 and 1.03 V when a 1 GΩ resistor was loaded, with the instantaneous peak power of 1.04 nW and power density of 1.52 µW kg^−1^ (Figure 6d,e). In contrast, the same PVDF membrane coupling with pure PVA showed no response with only background noise because of the insufficient driving force of humidity on PVA film to lift PVDF (Figure 6d,e). It is worth noting that the power generation here is supplied by natural evaporation without external manual humidity switching.

Due to the high sensitivity of the HCMOP‐6^d^ membrane to water, we fabricated flowerlike responsive actuators with five flower petals made by the HCMOP‐6^d^ membrane. When the flower was put above the water surface, the petals automatically closed in response to water vapor, and correspondingly, when the water stimulus was removed, the flowers bloomed again (Figure S29, Supporting Information). Moreover, the HCMOP‐6^d^ membrane could also be designed as an intelligent electricity switch. The HCMOP‐6^d^ membrane could bend upward on the water surface and push the copper wire upward to form a closed circuit, thus lighting the bulb (Figure 6b,c; Video S4, Supporting Information). A membrane‐based humidity switch was fabricated by connecting one end of the HCMOP‐6^d^ membrane to different colored LED lights, the circuit diagram is shown in Figure 6g. By varying the ambient humidity, the bending angles of the membrane can be immediately changed to form closed loops at different heights. Figure 6g shows that the LED lights with blue, green, and yellow colors were respectively controlled by the humidity of 27% RH, 50% RH, and 71% RH, which revealed potential applications in humidity detection.

## 3 Conclusion

In conclusion, we successfully synthesized a new boronic acid‐modified ZrMOP‐B(OH)_2_, which could generate dynamic boronic ester bonds with PVA polymer, hence affording a new type of covalently assembled hypercrosslinking network. A series of free‐standing and defect‐free HCMOP hybrid membranes were fabricated. Thanks to the dynamic reversibility property of boronic ester bonds triggered by humidity, the membranes showed interesting self‐healing properties. It was found that the self‐healing time became faster as increasing the humidity. In addition, ZrMOP‐B(OH)_2_ served as high connectivity nodes, which could significantly improve the membrane's mechanical properties, further promoting the durability of the membrane actuators. With the advantages of both porous MOPs and hygroscopicity PVA polymer, HCMOP membranes can realize humidity‐responsive actuating performances. Owing to rapid water adsorption and desorption, HCMOP could realize a self‐oscillating property above the water surface even after loading a cargo that was 1.5 times the weight of the membrane. Furthermore, we fabricated a new type of autonomous generator by coupling the HCMOP‐6 actuator with a piezoelectric PVDF membrane that could continuously transduce mechanical energy from self‐oscillating motions generated by water vapor into electricity, outputting voltage reached −0.93 and 1.03 V loaded the 1 GΩ resistor. This work provides a generation of smart materials and inspires the development of self‐oscillating materials for various applications.

## 4 Experimental Section

### Synthesis of MOPs

*ZrMOP‐B(OH)_2_*: H_2_BDC‐B(OH)_2_ (6.3 mg, 0.3 mmol) and Cp_2_ZrCl_2_ (17.5 mg, 0.6 mmol) were dissolved in 1 mL DMA and 100 µL water was added. After ultrasonication, the clear solution was thermally treated in a preheated oven at 60 °C for 8 h followed by 4 h cooling. Then, the colorless cubic single crystals were synthesized and washed with fresh DMA three times. The solvent exchange was carried out by soaking the crystals in acetone for 4 days, with the solvent being exchanged every 6 h. After that, ZrMOP‐B(OH)_2_ was dried under vacuum at 60 °C for 24 h.

*ZrMOP‐CH_3_*: A modified synthesis procedure was used for the synthesis of ZrMOP‐CH_3_.^[^ ^27^ ^]^ 2‐methylterephthalic acid (5.4 mg, 0.3 mmol) and Cp_2_ZrCl_2_ (17.5 mg, 0.6 mmol) were dissolved in 1 mL DMA and 0.5 mL MeOH and 100 µL water were added. After ultrasonication, the clear solution was thermally treated in a preheated oven at 60 °C for 8 h followed by 4 h cooling. Then, the colorless cubic single crystals were synthesized and washed with fresh DMA three times. The solvent exchange was carried out by soaking the crystals in acetone for 4 days, with the solvent being exchanged every 6 h. After that, ZrMOP‐CH_3_ was dried under vacuum at 60 °C for 24 h.

### Fabrication of Membranes

*PVA membrane*: PVA solution (20 wt.%) was prepared by dissolving PVA in water at 70 °C with stirring. The PVA solution was added into the PTFE mold, heated for 1 h in an oven at 60 °C, and the PVA film was carefully peeled off and dried overnight at 60 °C under vacuum, and then placed in a sample dryer.

*HCMOPs membrane*: The membrane fabrication procedure is shown in Scheme 1. According to the molar ratio of ZrMOP‐B(OH)_2_/PVA, a = 1/36, b = 1/18, c = 1/15, d = 1/12, ZrMOP‐B(OH)_2_ (HCMOP‐6^a^: 14.8 mg, HCMOP‐6^b^: 29.7 mg, HCMOP‐6^c^: 35.7 mg, HCMOP‐6^d^: 44.6 mg) first dissolved in 3–5 mL mixed solvent of DMF/H_2_O (10:3, v/v) to form a transparent aqueous solution, to which 20 wt.% PVA solution (0.4 g) was added. To promote polymerization, NEt_3_ (the molar amount of NEt_3_ is the same as that of ‐B(OH)_2_ group in ZrMOP‐B(OH)_2_) was added. The clear solution was poured on a PTFE mold. The PTFE mold was then covered with aluminum foil and dried at a certain temperature to completely evaporate the solvent. The membrane was peeled off from the PTFE mold and dried overnight at 60 °C under vacuum, and then placed in a sample dryer.

*ZrMOP‐CH_3_@PVA membrane*: Membranes of ZrMOP‐CH_3_@PVA were prepared via the same procedure as HCMOP‐6 membranes, replacing ZrMOP‐B(OH)_2_ with ZrMOP‐CH_3_ with the same molar amount.

### Synthesis of Model Molecules

*Model molecule (1)*: To a solution of phenylboric acid (0.61 mg, 5 mmol) and propylene glycol (1 mL, 13.6 mmol) in mixed solvent DMF/H_2_O (1 mL, 10/3, v/v) stirred was added NEt_3_ (0.51 g, 5 mmol). The resulting reaction mixture was stirred at room temperature overnight. The mixture was concentrated in a vacuum and dried over CaCl_2_. Then the mixture was added to 5 mL ether, filtered, and concentrated in a vacuum. Silica gel column chromatography (hexane/ethyl acetate = 4/1, v/v) gave the target product light yellow oil.

*Model molecule (2)*: ZrMOP‐B(OH)_2_ reacted with propylene glycol under the same solution conditions as the preparation of the HCMOP‐6^d^ membrane. The mixture was stirred overnight at room temperature, and the solvent in the system was removed in an oven to obtain a white powder. The unreacted propylene glycol was washed with a small amount of dichloromethane.

### Preparation of Generators

The 25 µm commercial piezoelectric PVDF film deposited with the aluminum electrode on both sides was purchased from Jinzhou KEXIN Electronic Material Co., Ltd. First, stick an insulating PI tape on one side of the piezoelectric PVDF film to prevent the upper and lower aluminum electrodes from contacting during the operation. Copper foils were attached on both sides of PVDF by PI tape as electrodes to connect with the test instrument. The upper and lower ends of the actuator were adhered to the piezoelectric PVDF film with two very thin double‐sided tapes to prepare the generator.^[^ ^46^, ^47^ ^]^

### Performances Evaluation Procedure of Actuators

Actuators or pure polymer membranes of the same sizes were vertically placed on the water surface (the screen will not touch the water surface), and the water surface height was fixed each time, the camera was used to record the water vapor drive process of each film. The performance of each actuator was evaluated by the blending angle of different actuators.

### Performances Evaluation Procedure of Generators

The actuator was coupled with a commercial piezoelectric PVDF (Aluminum plated, 25 mm × 15 mm), which was connected with Keithley 2450 (Keithley Instruments, the circuit parameter of open‐circuit voltage test was set current to 0 A) to monitor its voltage output (the circuit diagram is shown below). A glass of water was placed under the actuator and across the screen. HCMOP‐6^d^ drives piezoelectric PVDF self‐oscillating motion on the water surface without additional operation (On the day of the test, the temperature was ≈18 °C, the air humidity was ≈25% RH, and the water temperature was ≈25 °C). The motion of the generator during the whole process was recorded by a camera.

## Conflict of Interest

The authors declare no conflict of interest.

## Supporting information

Supporting Information

Supplemental Video 1

Supplemental Video 2

Supplemental Video 3

Supplemental Video 4

## Data Availability

The data that support the findings of this study are available in the supplementary material of this article.
