# Supplementary material for: Humidity‐Induced Self‐Oscillating and Self‐Healing Hypercrosslinked Metal–Organic Polyhedra Membranes
Source: Adv Sci (Weinh). 2024 Mar 11;11(20):2307376. doi: 10.1002/advs.202307376 (PMC11132063; doi:10.1002/advs.202307376)
Supplement: Supplementary file 1 — Supporting Information [file ADVS-11-2307376-s003.pdf]

ADVANCED SCIENCE
Open Access

Supporting Information

for *Adv. Sci.*, DOI 10.1002/advs.202307376

Humidity-Induced Self-Oscillating and Self-Healing Hypercrosslinked Metal–Organic Polyhedra Membranes

*Jiamin Li, Zhaoyi Liu, Jinjin Liu, Xue Liu, Yang Luo, Jiajie Liang and Zhenjie Zhang**

Supporting Information

Humidity-Induced Self-Oscillating and Self-Healing Hypercrosslinked Metal-Organic Polyhedra Membranes

*Jiamin Li,[#] Zhaoyi Liu,[#] Jinjin Liu,[#] Xue Liu, Yang Luo, Jiajie Liang, and Zhenjie Zhang**

Table of Contents

S1. Details on experimental methods..2
S2. Supplementary figures..3
S3. Supplementary table...17

S1. Details on experimental methods

General

Chemicals and solvents were purchased from commercial sources and used as received without further purification. The type of polyvinyl alcohol (PVA) is PVA 1788.

Characterization

Nuclear magnetic resonance spectroscopy (NMR): ^{1}H NMR spectra were recorded on Bruker AV400 instruments at 400 MHz. Chemical shifts were reported in parts permillion (ppm) down field from internal tetramethylsilane.

Powder X-ray diffraction (PXRD) measurements: PXRD was recorded on a D/Max-2500 X-ray diffractometer using Cu-Kα radiation by depositing powder on a glass substrate, 2θ from 2° to 30° with 1° increment.

Ultra performance liquid chromatography-quadrupole time-of-flight mass spectrometry (UPLC-Q-TOF-MS): UPLC-Q-TOF-MS was performed using Waters ACQUITY UPLC I-Class/UPCC/M-Class/SYNAPT G2-SI.

Gas sorption measurements: Adsorption measurements were performed using a Micromeritics ASAP 2460. Before gas sorption tests, synthesized MOPs were soaked in acetone (refreshed every 12 h) for 5 d. Approximately 60-100 mg of each MOP were transferred to preweighed sample tubes and evacuated on a vacuum line for ∼1 h at room temperature, and subsequently degassed at room temperature on an ASAP 2460 adsorption analyzer for 10 h. The sample tube was reweighed to obtain a consistent mass for the degassed sample. N_2 adsorption isotherms were measured at 77 K; the temperature was held constantly using liquid nitrogen.

Fourier transform infrared (FT-IR): All FT-IR spectra were recorded on a Nicolet iS 50 ATR-FTIR instrument.

Scanning electron microscopy (SEM) and energy-dispersive x-ray (EDX): SEM images and EDX were taken with Hitachi JSM-7500F scanning electron microscopy.

Field Emission Scanning electron microscopy (FE-SEM): High magnification SEM images were taken with JEOL JSM-7800F scanning electron microscopy.

Stress−strain curves: The stress and strain curves were measured using a MArk-10 SeriesF series advanced mechanical machine with IntelliMESUR® software.

Water vapor adsorption-desorption and dynamic water adsorption (DVS): Water vapor adsorption-desorption experiments were performed using BSD-VVS (BeiShiDe Instrument) at 298 K from 0% to 95% (dry nitrogen as the carrier).

Shear sweep measurement: The rheological behavior of the solution was evaluated at 25 ℃ using a DHR-2 rheometer (TA Instruments) with a 20 mm parallel plate geometry and 1000 μm gap.

Small-angle X-ray scattering (SAXS): SAXS patterns were collected on a Rigaku NANOPIX system with an exposure time of 600 s for one measurement. The X-ray generator was Fr-x (rotation anode X-ray generator) with a power of 2.97 kW (45 kV, 66 mA) and a target/wavelength of Cu/1.54018 Å. The camera length was 170 mm. The incident beam was in New CMF optics, and the detector was HyPix-54000.

S2. Supplementary figures

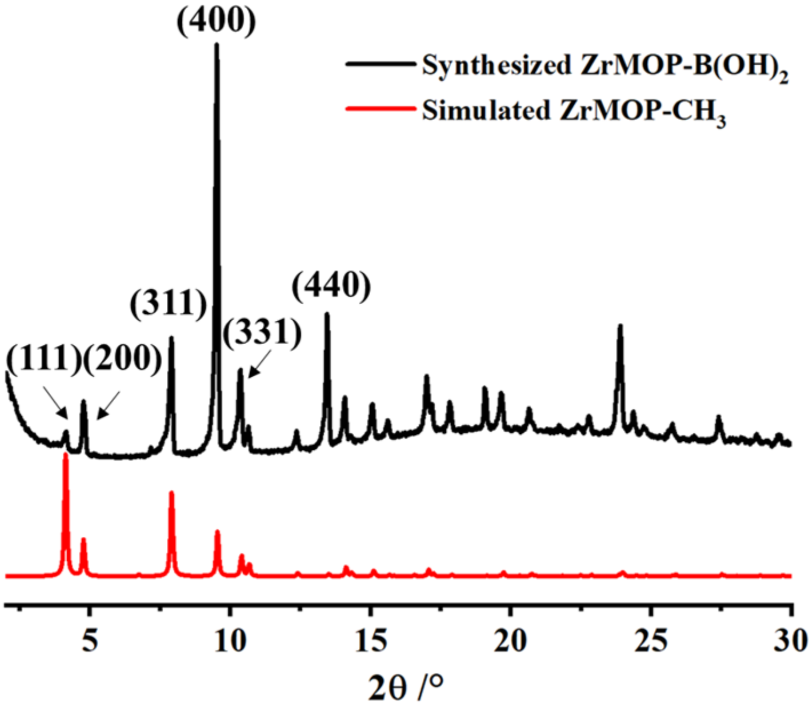

Figure S1. PXRD patterns of synthesized ZrMOP-$B(OH)_2$ crystal compared to the simulated pattern of ZrMOP-CH_3.

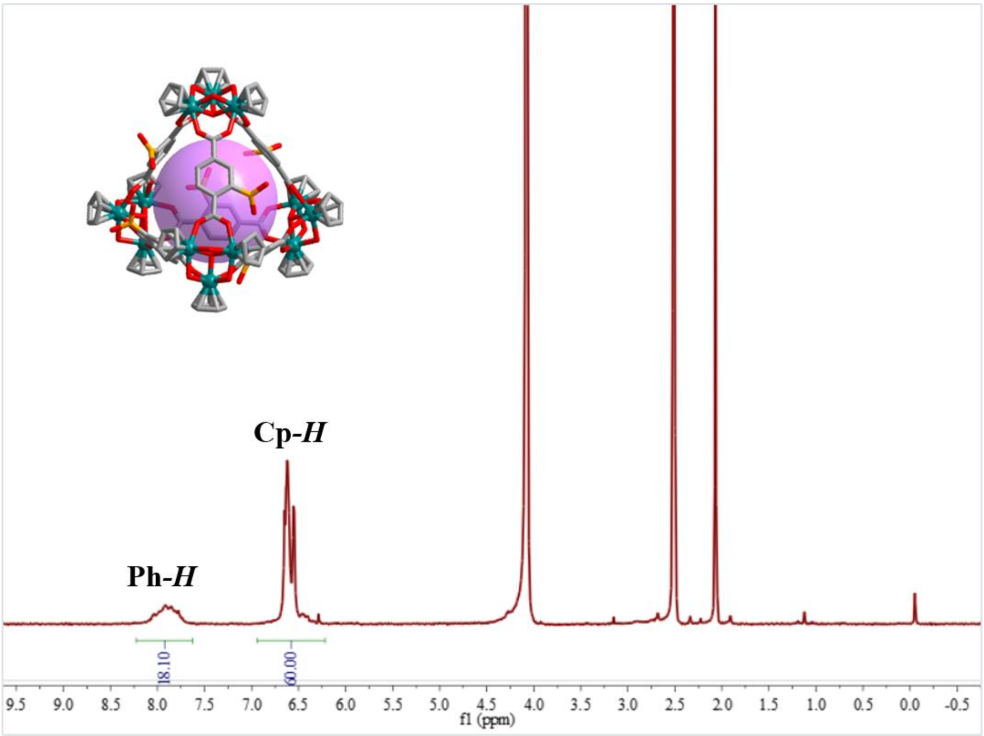

Figure S2. ^{1}H NMR spectra of ZrMOP-B$(OH)_2$ in DMSO-d_6/D_2O.

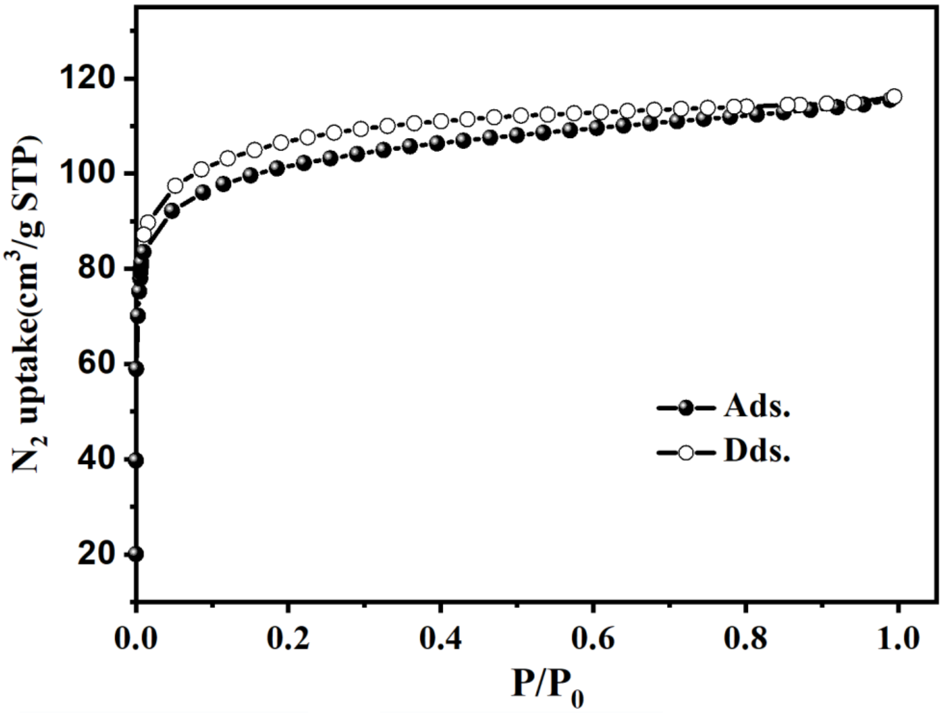

Figure S3. N_2 adsorption analysis of ZrMOP-B$(OH)_2$ at 77K.

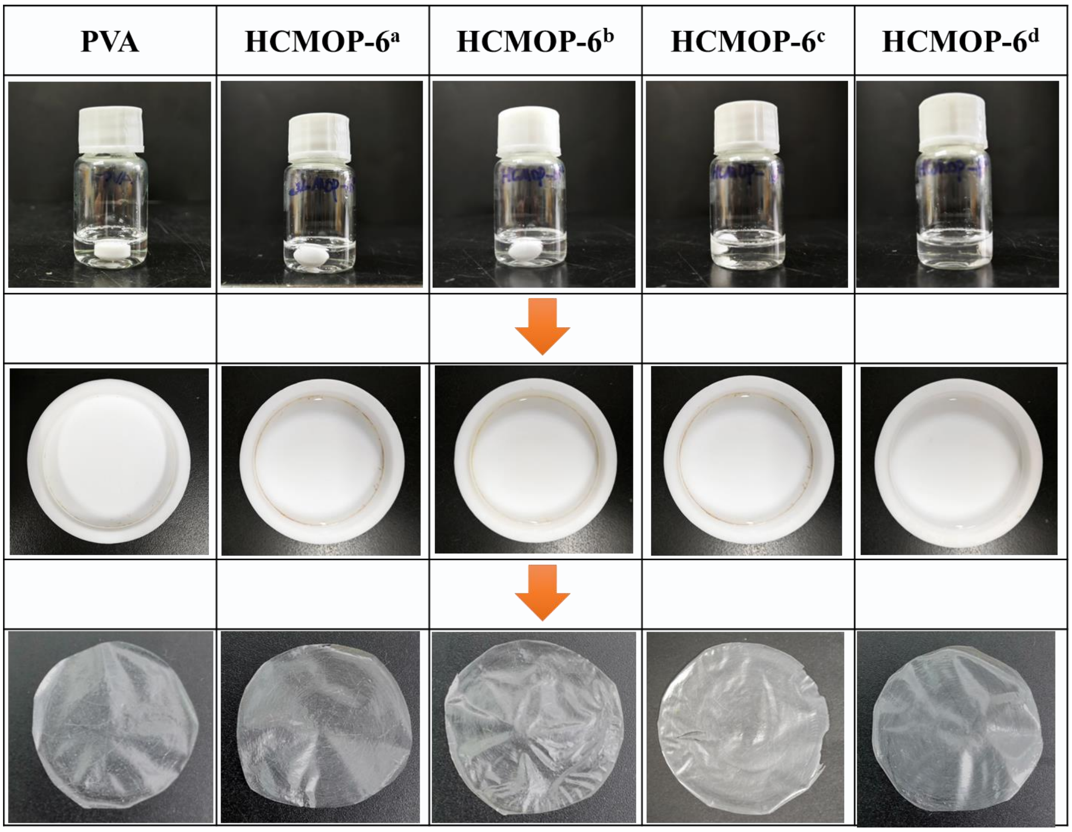

Figure S4. The photographs of PVA membrane and HCMOPs with different amounts of MOPs. All samples can form continuous membranes after the solvent evaporates.

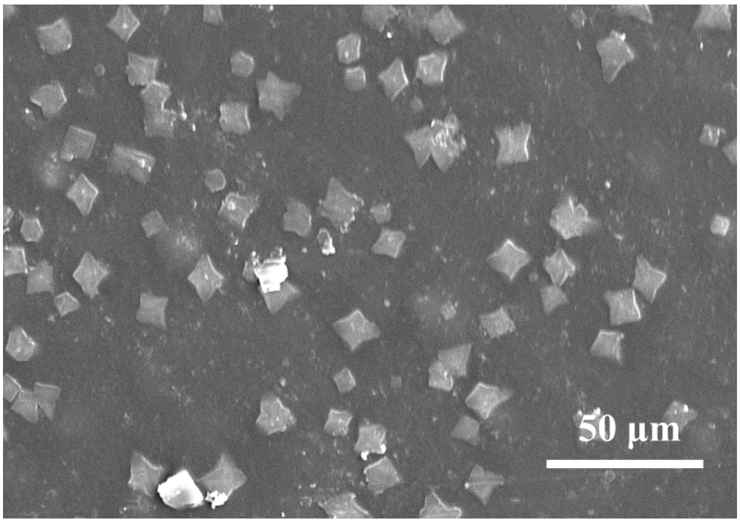

Figure S5. Top view SEM image of HCMOP membrane with ZrMOP-B$(OH)_2$/PVA = 1/9 molar ratio.

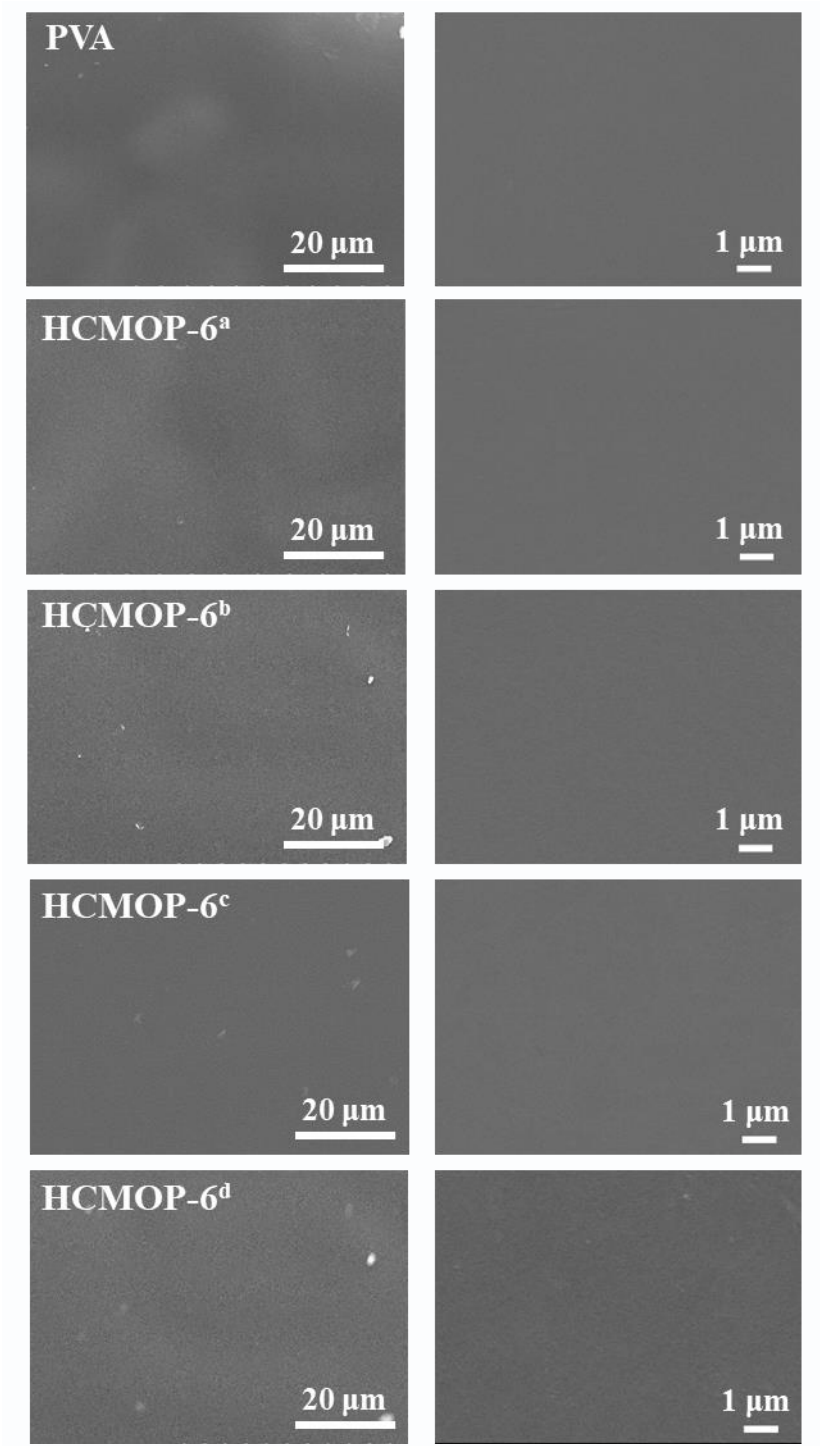

Figure S6. Top view SEM images of the freestanding membranes**.**

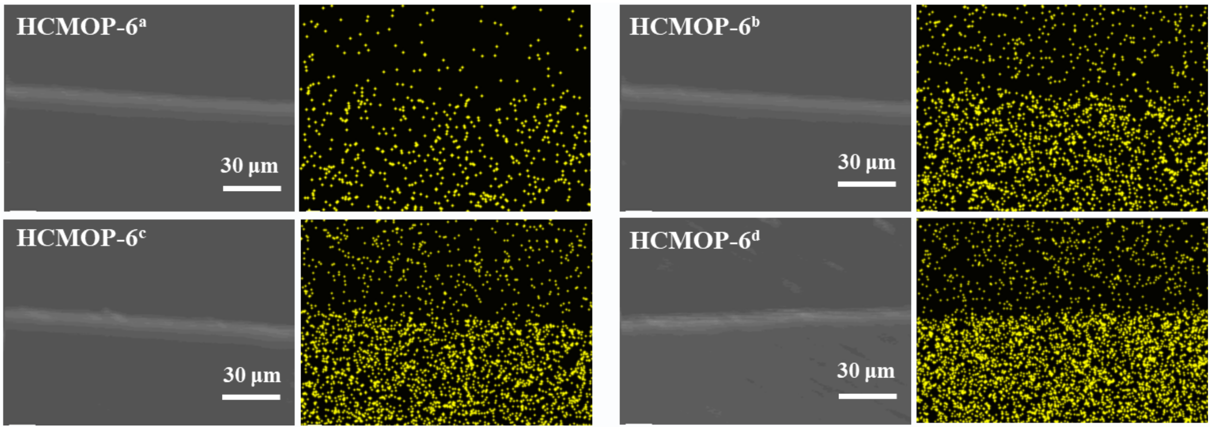

Figure S7. SEM-EDX mapping showing the Zr signal localized in HCMOP-6 membranes.

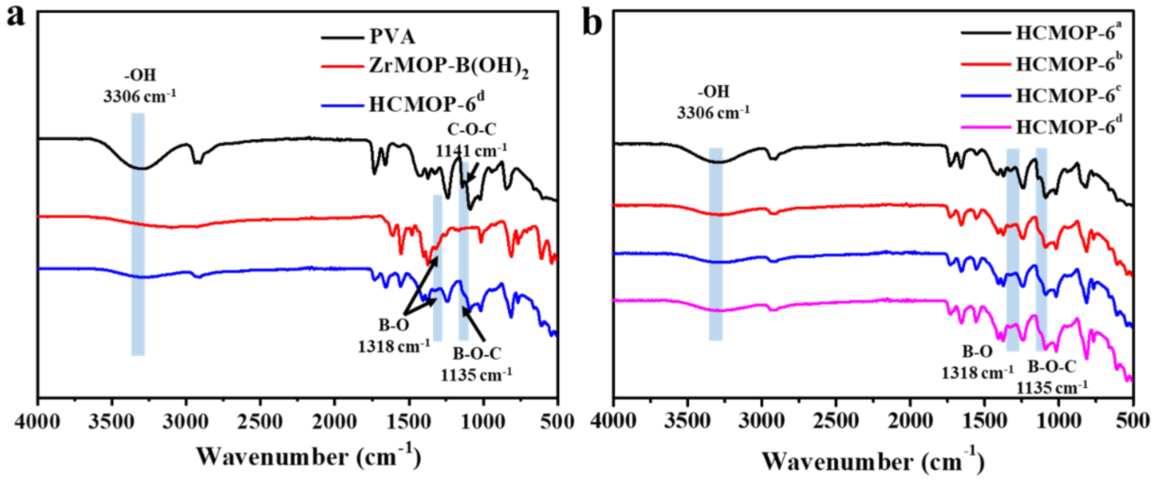

Figure S8. FT-IR spectra of PVA, $ZrMOP-B(OH)_2$ and HCMOP-6 membranes.

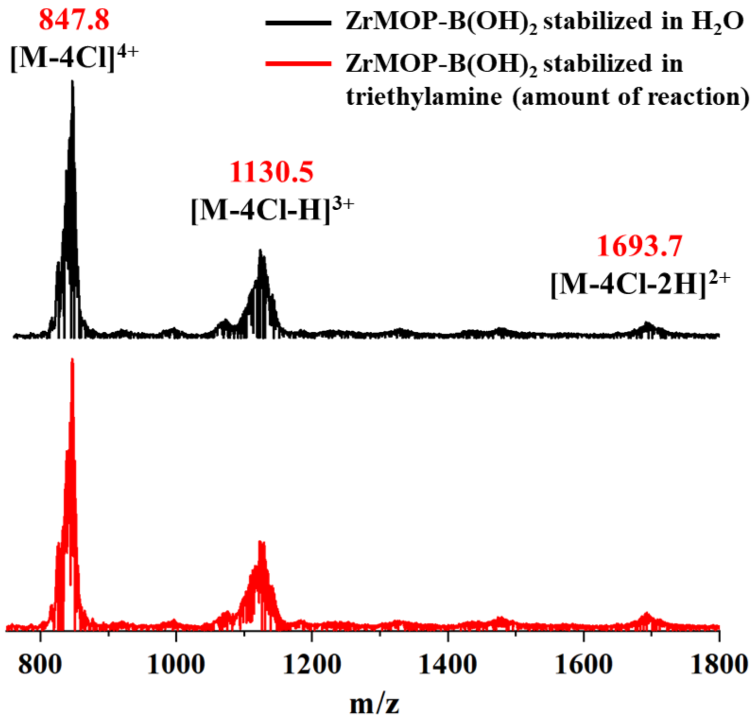

Figure S9. UPLC-Q-TOF-MS analysis of the stability of ZrMOP-B$(OH)_2$ in DMF under the existence of H_2O or triethylamine (amount of reaction).

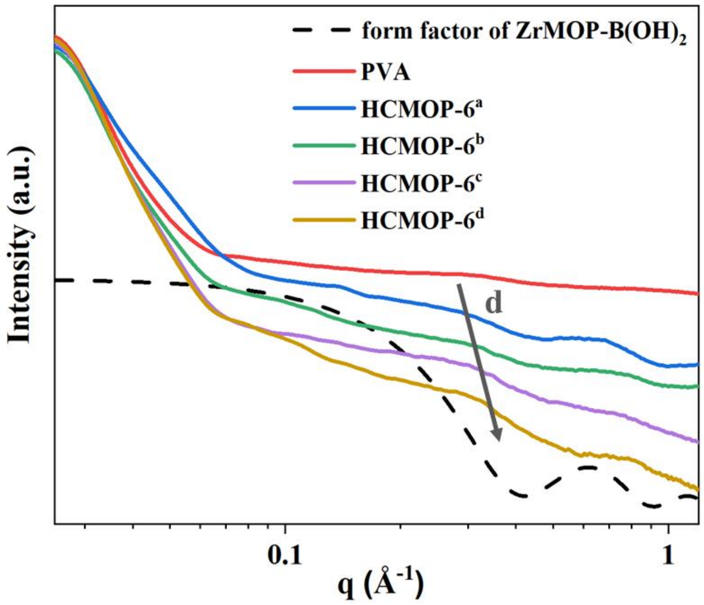

Figure S10. SAXS for PVA and HCMOP-6 membranes (thickness: ~20 μm). The black dotted line is the theoretical SAXS curve of the ZrMOP-B$(OH)_2$ generated from its single-crystal diffraction structure model. d is the average distance between neighboring MOPs in the polymer matrix.

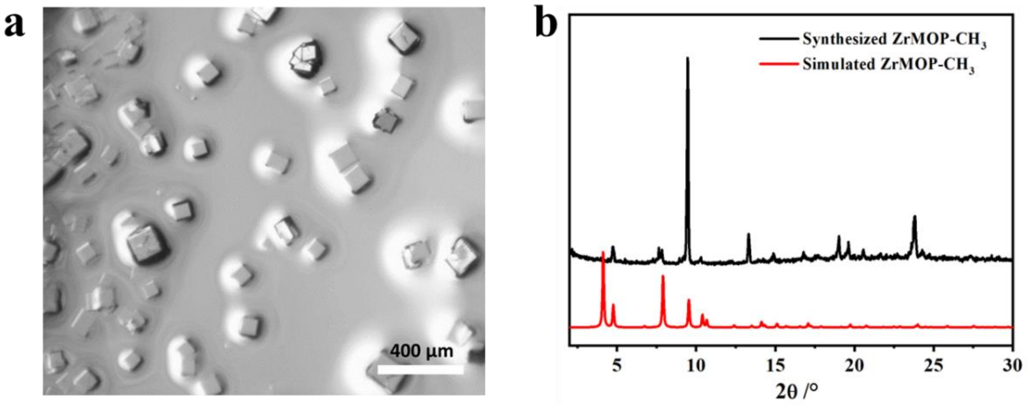

Figure S11. a) Optical microscope photo of $ZrMOP-CH_3$ crystals. b) Simulated and synthesized PXRD patterns of $ZrMOP-CH_3$.

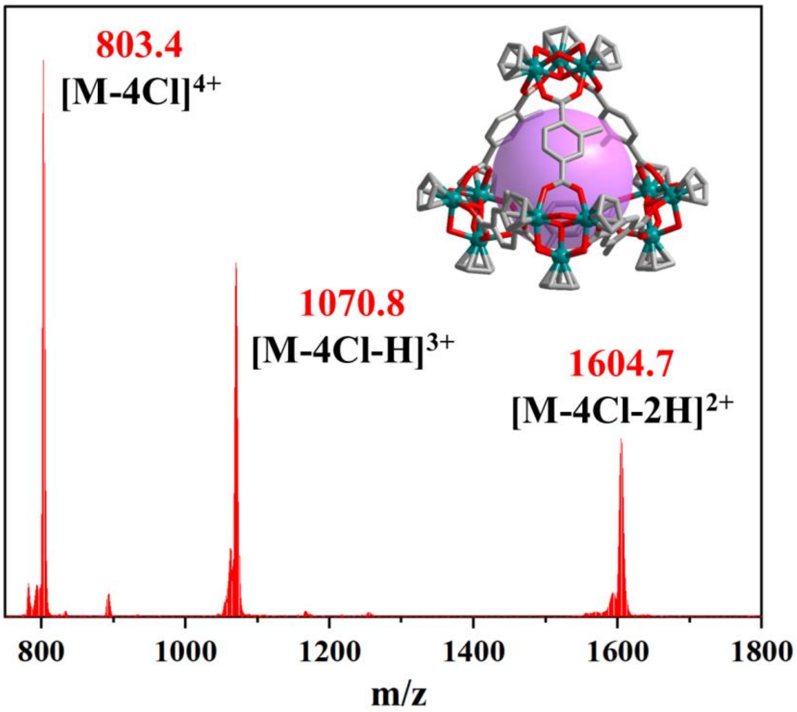

Figure S12. UPLC-Q-TOF-MS analysis of $ZrMOP-CH_3$ in methanol.

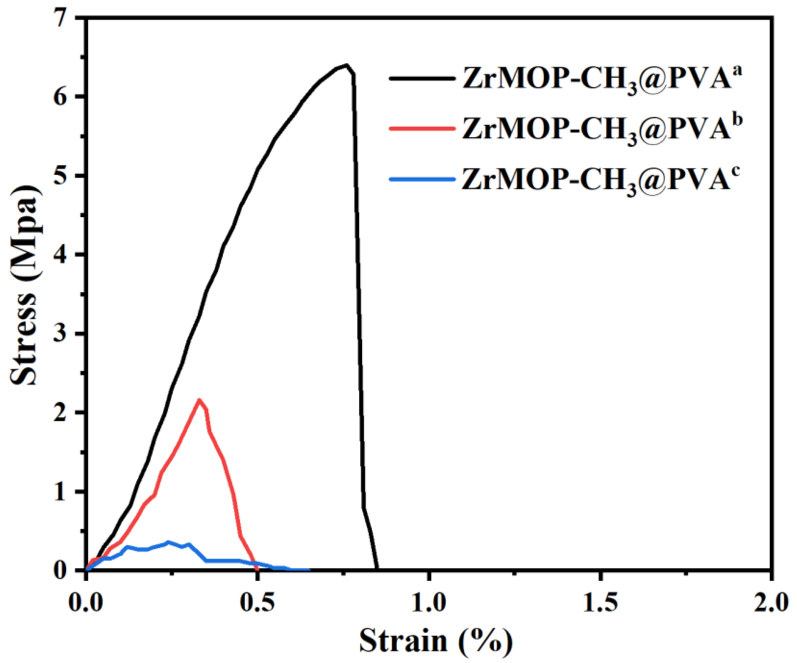

Figure S13. Stress−strain curves of ZrMOP-CH_3@PVA membranes. ZrMOP-CH_3@PVA[d] is N/A due to its extremely poor mechanical performance.

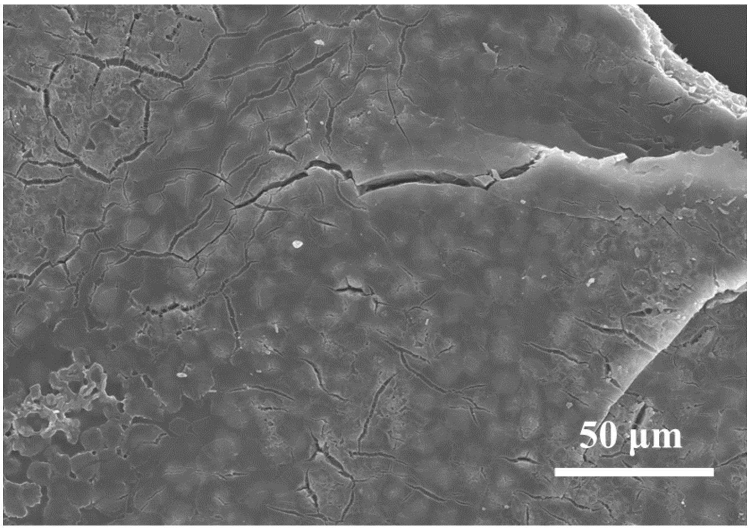

Figure S14. SEM image of ZrMOP-CH_3@PVA[d] membrane.

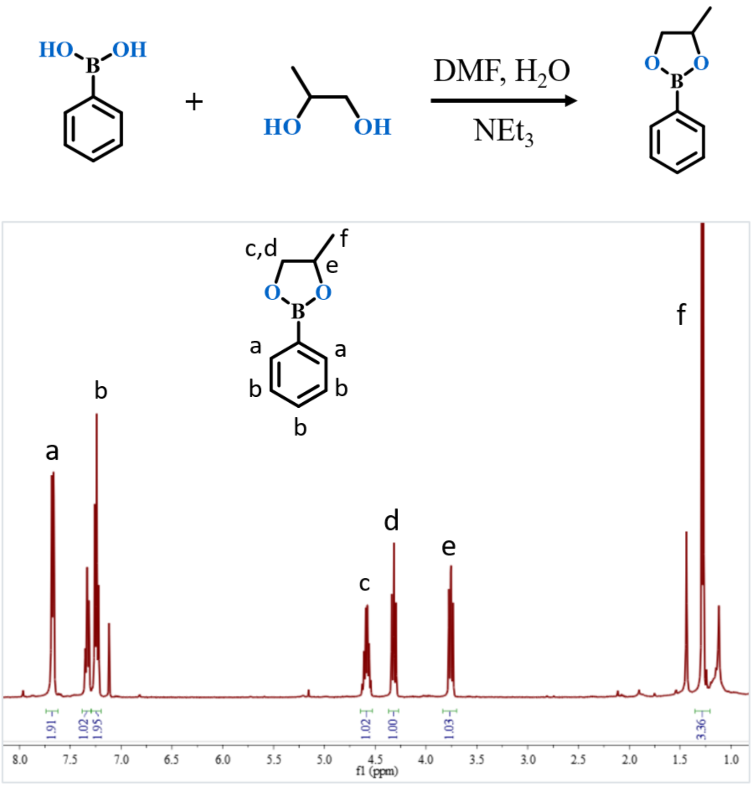

Figure S15. ^{1}H NMR spectra of model molecule (1) in $CDCl_3$.

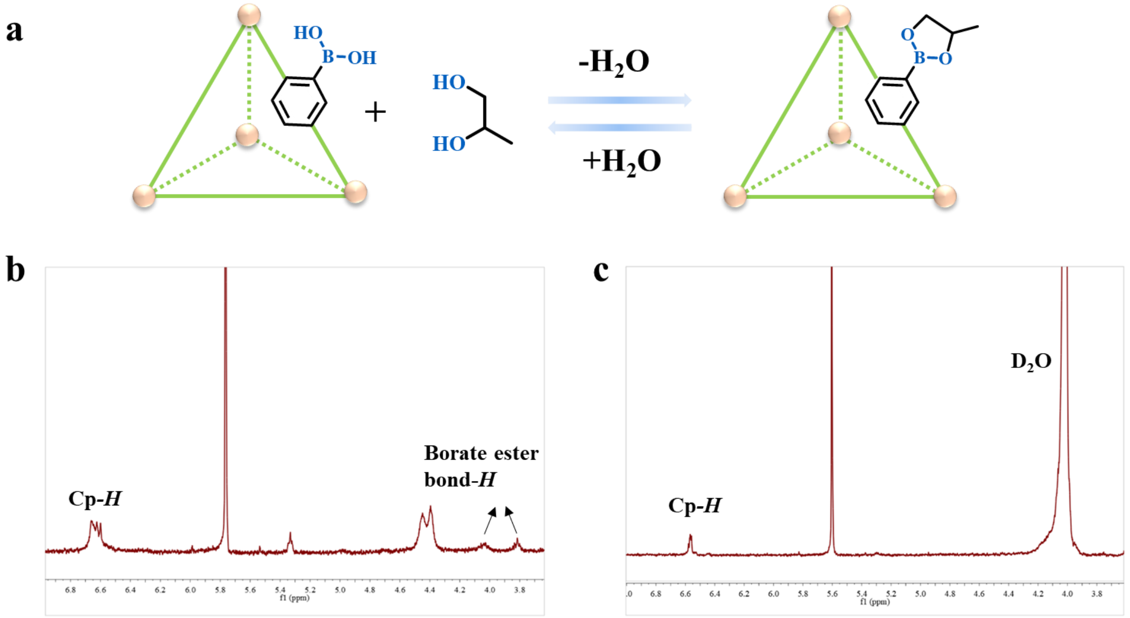

Figure S16. a) The schematic diagram of ZrMOP-$B(OH)_2$ reacted with propylene glycol (model molecule (2)). b) ^{1}H NMR spectra of model molecule (2) in DMSO-d_6. c) ^{1}H NMR spectra of model molecule (2) with the addition of D_2O in DMSO-d_6.

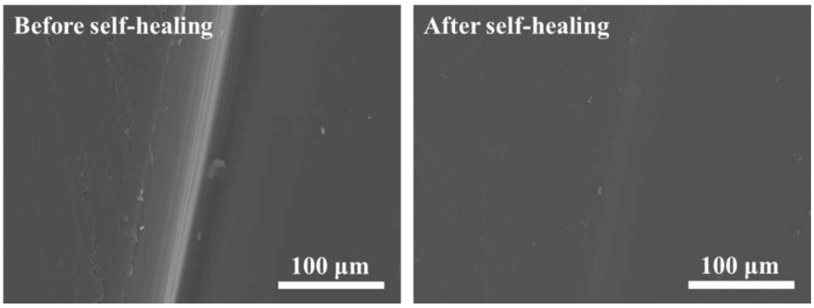

Figure S17. SEM images of damaged HCMOP-6[d] membrane and self-healed HCMOP-6[d] membrane.

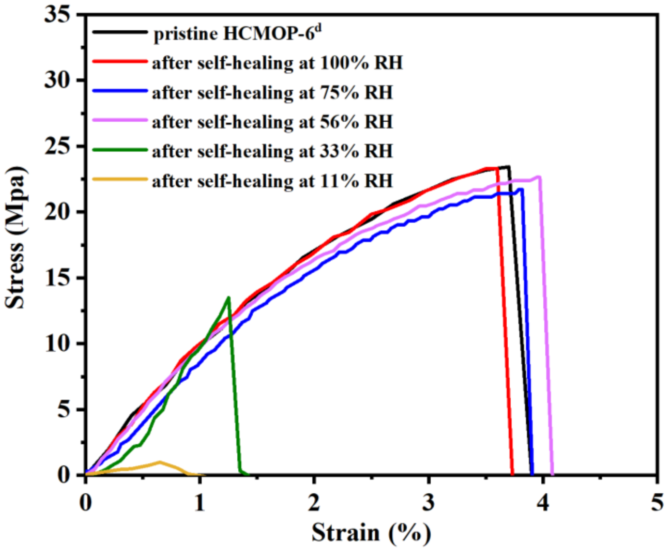

Figure S18. Stress–strain curves of HCMOP-6[d] membranes self-healed at different humidities.

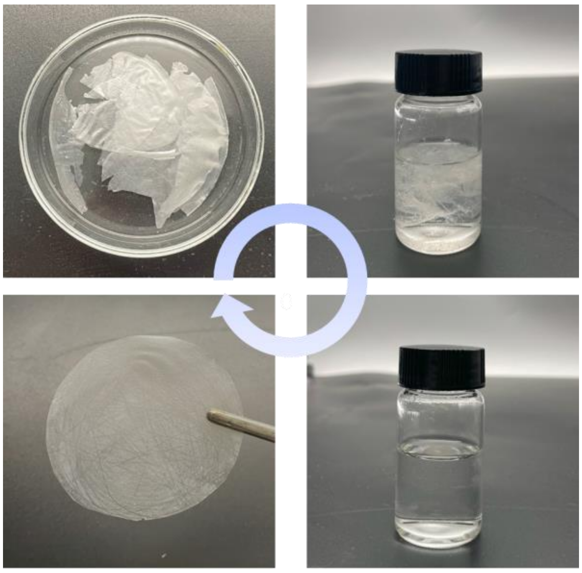

Figure S19. Reprocessing of the HCMOP-6[d] membrane through solvent processing in DMF/H_2O mixed solution under heating at 60 °C (thickness: ~20 µm).

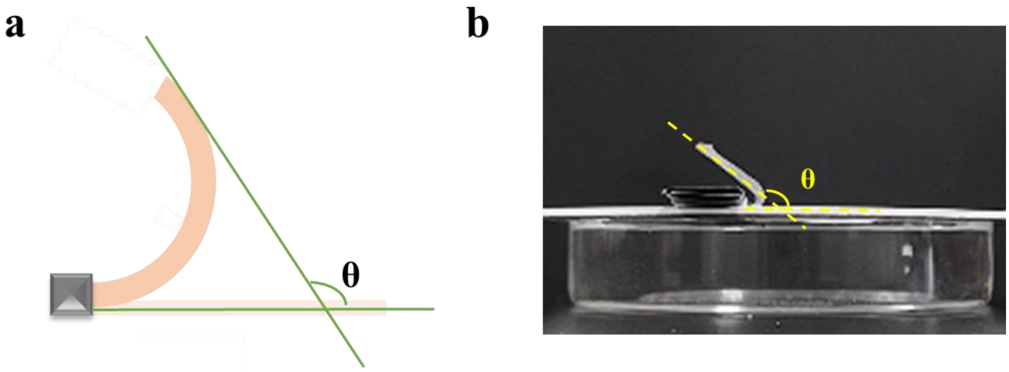

Figure S20. a) The schematic diagram of the definition and measurement of the bending angle. b) The actual diagram of the definition and measurement of the bending angle.

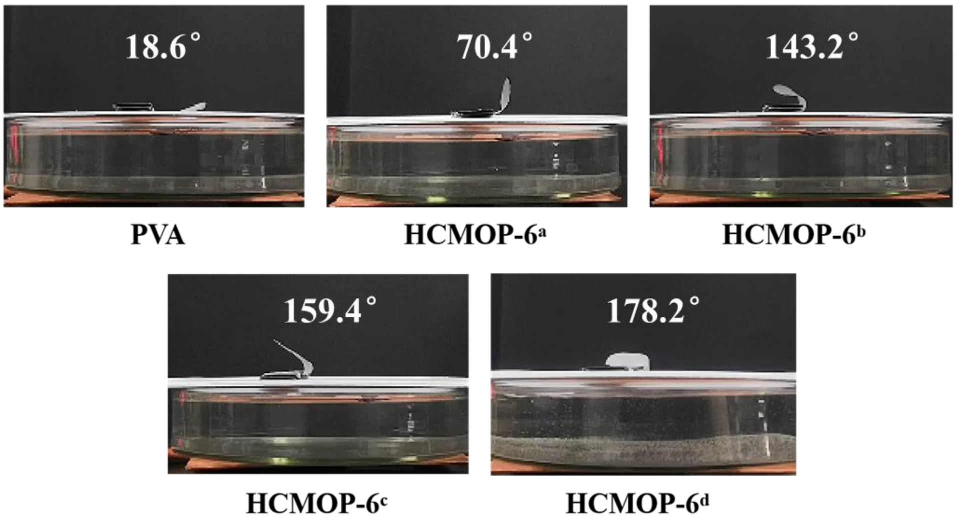

Figure S21. Photographs of PVA and HCMOP-6 bent above water surface for 10 seconds (thickness: 20 μm, ambient environment: 25 ℃, ~18% RH, water temperature: 25 ℃).

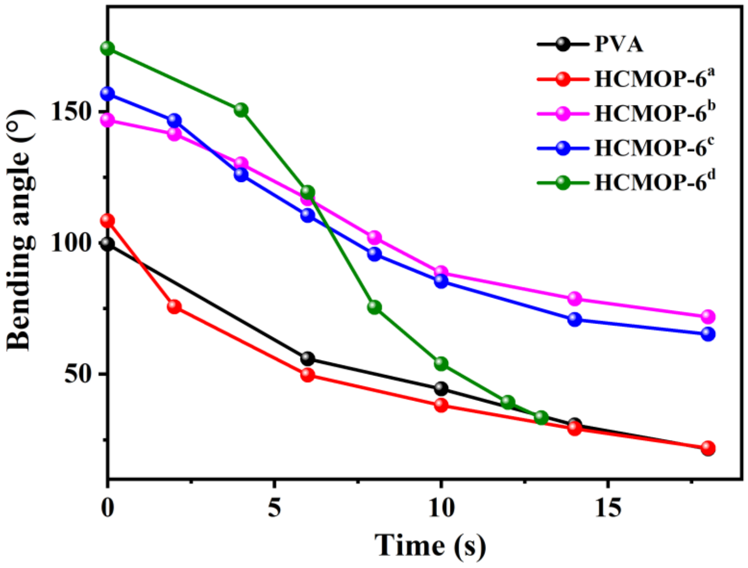

Figure S22. Comparison of recovery angle of PVA and HCMOP-6 membranes upon water desorption (thickness: 20 μm, ambient environment: 25 °C, ~18% RH, water temperature: 25 °C).

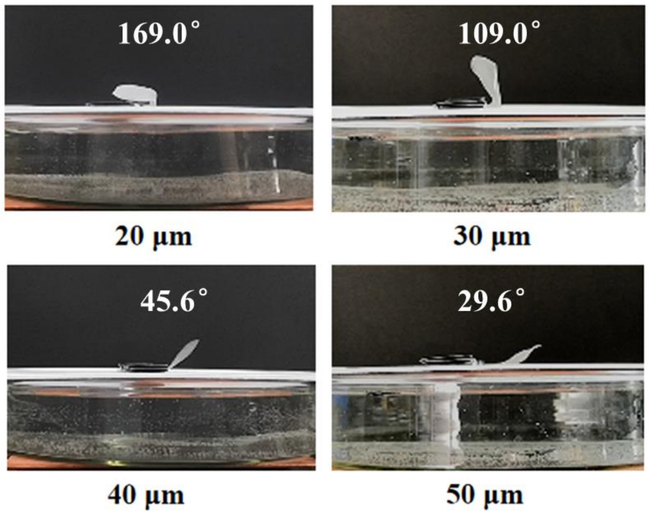

Figure S23. Photographs of HCMOP-6[d] with different thicknesses bent above water surface for 8 seconds (ambient environment: 25 °C, ~20% RH, water temperature: 25 °C).

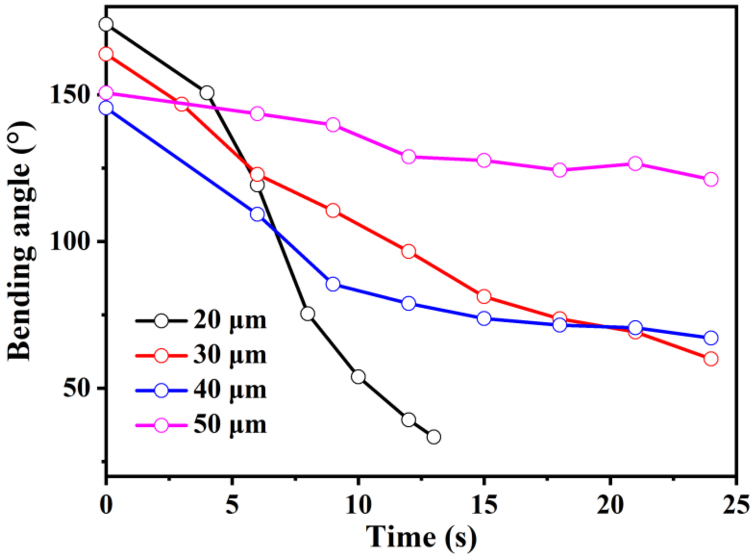

Figure S24. Comparison of recovery angle of HCMOP-6^d with different thicknesses upon water desorption (ambient environment: 25 °C, ~20% RH, water temperature: 25 °C).

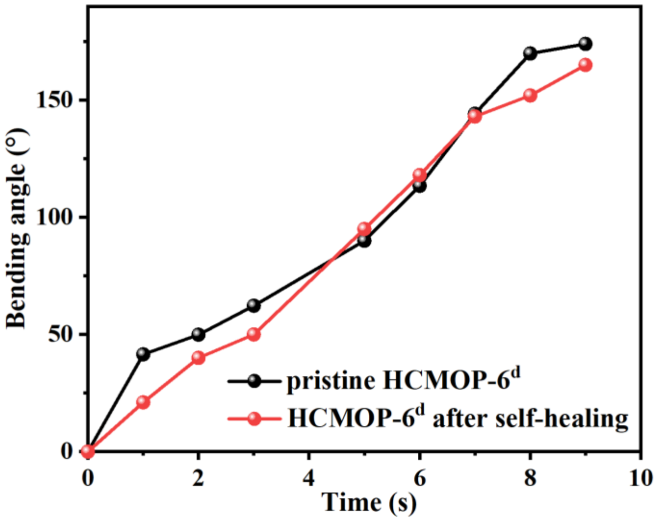

Figure S25. Bending angle of pristine HCMOP-6^d and the sample after self-healing under humidity stimulation (thickness: 20 μm, ambient environment: 25 °C, ~18% RH, water temperature: 25 °C).

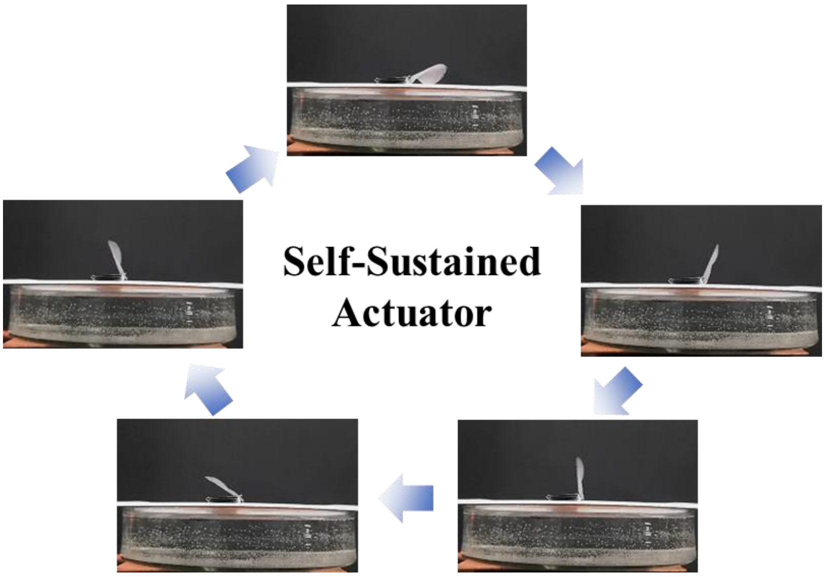

Figure S26. Photographs of the self-oscillation experiment of HCMOP-6[d] above the water surface (thickness: 20 μm, ambient environment: 25 °C, ~20% RH, water temperature: 25 °C).

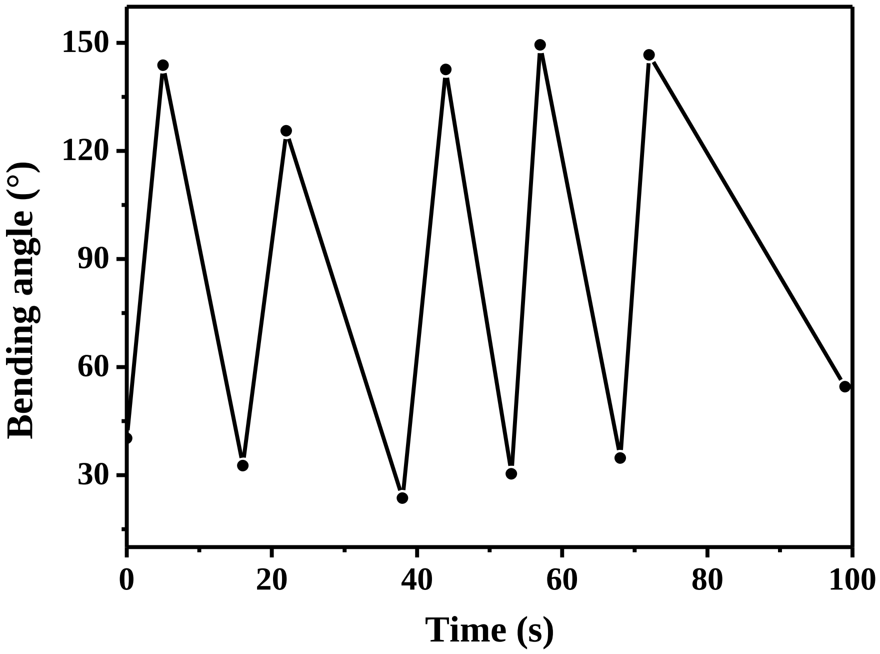

Figure S27. The loading-self-oscillation process of HCMOP-6[d] underwent 5 cycles upon moisture gradients on/off switches (thickness: 20 μm, ambient environment: 25 °C, ~20% RH, water temperature: 25 °C).

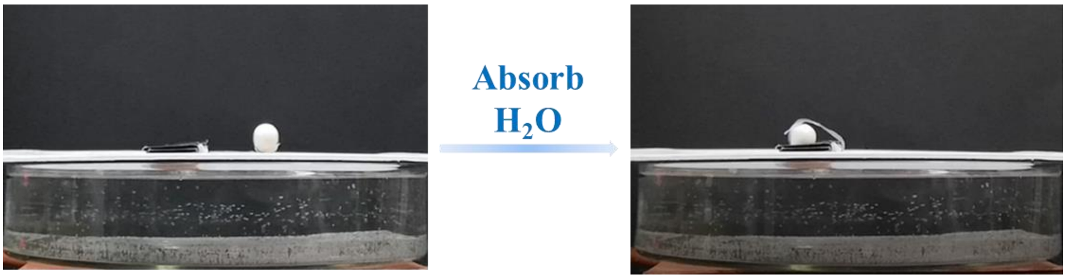

Figure S28. Photographs of HCMOP-6[d] bending with cargo above the water surface (the weight of the cargo is 10 times that of the membrane itself but the membrane cannot return) (thickness: 20 μm, ambient environment: 25 °C, ~20% RH, water temperature: 25 °C).

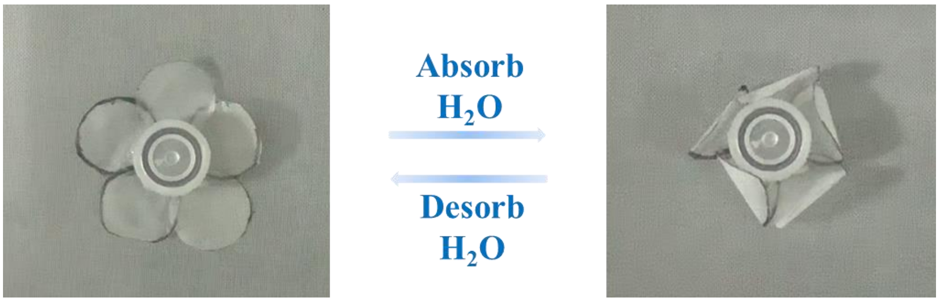

Figure S29. Photograph of the flowering/closing demonstration of HCMOP-6[d] above the water surface (thickness: 20 μm, ambient environment: 25 °C, ~20% RH, water temperature: 25 °C).

S3. Supplementary table

Table S1. Mechanical performance analysis of PVA and HCMOP-6 membranes.

Name	Young's modulus (MPa)	Ultimate stress (MPa)	Strain at break (%)
PVA	156.8±9.5	12.6±2.3	6.6±0.4
HCMOP-6[a]	336.0±8.8	16.0±2.5	5.7±0.5
HCMOP-6[b]	433.5±8.1	17.3±1.6	5.0±0.7
HCMOP-6[c]	588.6±6.2	22.2±2.5	4.2±0.4
HCMOP-6[d]	815.3±8.6	25.1±2.5	3.9±0.5
